# Thinking afresh about the classification of eating disorders

**DOI:** 10.1002/eat.20460

**Published:** 2007-09-14

**Authors:** Christopher G Fairburn, Zafra Cooper

**Affiliations:** Oxford University Department of PsychiatryOxford, United Kingdom

The DSM-IV scheme for classifying eating disorders^1^ has certain fundamental flaws. Most prominent among them is the fact that the supposedly “residual” diagnostic category, eating disorder not otherwise specified (EDNOS), is the most common eating disorder encountered in clinical practice: it is more common than the two specified eating disorders, anorexia nervosa (AN) and bulimia nervosa (BN). This is firmly established among adults who are outpatients,^2^–^7^ and now Rockert et al. have demonstrated that this is true of those attending a tertiary care outpatient center.^8^ It also seems to be the case in inpatient settings^9^ and among adolescent cases.^10^,^11^

A second problem concerns the utility of the current diagnostic distinctions. Their arbitrariness is immediately apparent when working with patients. This is especially obvious when one follows patients over time as migration between the eating disorder diagnoses is the norm rather than the exception. Many cases of AN evolve into BN^12^ or EDNOS, and many cases of BN evolve into EDNOS.^13^,^14^We suggest that these changes in clinical state do not truly reflect recovery from one psychiatric disorder and the development of another, as the DSM-IV scheme would suggest, but rather the evolution of a single eating disorder. If we were to tell those patients with EDNOS who have a history of AN and BN that they have had three different psychiatric disorders, two of which they have recovered from, they would react with skepticism. Perhaps we, as clinicians, should do the same.

With these flaws in mind, we suggest that there is a need to think afresh about the classification of eating disorders. Specifically, we suggest that we need answers to the following two overarching questions:

1. What is an “eating disorder”?
2. What are the grounds for subdividing the overall category eating disorder?

In addition, there is the pragmatic question of what to do about the classification of eating disorders while we are seeking answers to these questions.

## What is an “Eating Disorder”?

This would seem to be a basic question to ask when considering the classification of eating disorders, yet it is barely discussed. An answer to this question is needed to decide whether a problem with eating is sufficiently severe to constitute a “case”. Having a definition of eating disorder “caseness” would be of great value for epidemiological purposes, for evaluating response to treatment, and for clarifying the relationship between obesity and an eating disorder. This issue is highlighted by Devlin’s paper,^15^ “Is there a Place for Obesity in DSM-V?” in which he argues that obesity is an outcome (he calls it a state) and that his question can best be answered by considering the process of “nonhomeostatic overeating” that contributes to it. He notes that a certain degree of such overeating now appears to be the norm in our culture and as such should not be considered a psychiatric disorder. Thus the challenge becomes one of investigating whether there is some form of “non-normative” overeating leading to obesity which is best understood as a psychological or behavioral disorder. In attempting to meet this challenge Devlin considers, among other approaches, whether this form of overeating should be seen as an eating disorder, particularly focusing his discussion on binge eating disorder (BED) and night eating syndrome. It is of note that neither of these categories is formally recognized as yet and that Devlin’s discussion immediately raises the question of the definition of an eating disorder and the related question (see below) of the basis on which diagnostic subdivisions should be made.

In their everyday practice, clinicians must in fact have some implicit notion of what constitutes a case. Without such a notion it would at present only be possible to diagnose AN and BN since it is only these disorders that have specified diagnostic criteria. There are no diagnostic criteria for EDNOS, the diagnosis being reserved for patients who have an eating disorder of clinical severity that does not meet the diagnostic criteria for AN or BN.

Despite the fundamental importance of having a definition of an eating disorder few have been proposed, let alone operationalized. Fairburn and Walsh^16^ proposed the following definition. “A persistent disturbance of eating behavior or behavior intended to control weight, which significantly impairs physical health or psychosocial functioning.” This definition is consistent with the DSM-IV definition of a mental disorder, which requires that there be “clinically significant impairment or dis-tress.”^1^ The impairment requirement is crucial for it is the presence of this that determines the boundary between being a case or non-case.

To operationalize the definition of an eating disorder therefore requires determining which eating disorder features (and what levels) result in clinically significant impairment. To date we are aware of only one attempt to do this.^17^ More are needed.

## On What Basis Should Diagnostic Subdivisions be Made?

The second question concerns whether there are useful subdivisions to be made within the overall category eating disorder. This raises another fundamental but sometimes neglected question, “Why do we make diagnoses?” In other words, “What is the purpose of the DSM classificatory scheme?” This is an important question to keep in mind for there is no single “right” way of classifying things. If one takes plants, we might think that the scheme pioneered by Linnaeus is the definitive one, but while it is suitable for some purposes it is not for others. It is of little value to flower arrangers or vegetarians who will both wish to classify plants quite differently.

The purpose of clinical classificatory schemes such as DSM and ICD is to aid clinical work and, most importantly, to provide guidance regarding differences in treatment response and prognosis.^18^ As Kendell^19^ succinctly put it, “In the last resort all diagnostic concepts stand or fall by the strength of the prognostic and therapeutic implications they embody.” The question, therefore, is whether there are empirically supported divisions to be made within the category “eating disorder” in terms of differential treatment response or outcome. Surprisingly little is known about this. The existing three diagnoses have not been validated in this regard, and nor have any of the proposed new categories, a possible exception being BED which does seem to have a different course and prognosis from BN.^14^ Here there may be grounds for a distinction.

There is a tendency to derive new ways of classifying eating disorders simply on the basis of current psychopathology. While doing this might possibly point to clinically meaningful differences, there is a risk of creating distinctions that do not have clinical importance in terms of treatment or prognosis. Thus it is vital for such studies to include data on treatment and prognosis. It is perhaps worth remembering that moths of the same species (e.g., *Biston betularia*) can look remarkably different from each other yet behave in the same way.

In their report on a tertiary care sample Rockert et al.^8^ divided their EDNOS cases into six subgroups derived on an a priori basis. While some interesting differences emerged, it is not clear whether these differences were clinically meaningful. It would, therefore, be of great interest if Rockert et al. were to report the relative outcomes of these patients. Mitchell et al.^7^ took a different approach in that they used latent profile analysis to derive subgroups within EDNOS. Again there were some interesting differences between the subgroups, but once more it is not clear whether they were of any clinical consequence. It would be of interest to repeat this statistical exercise with the entire patient sample thereby putting aside the current (unvalidated) diagnostic distinctions, but of even greater interest would be the inclusion in the analyses of data on course or outcome.

What is termed “purging disorder” is the latest example of the subdivision of eating disorders on the basis of current psychopathology alone. As Keel^20^ stresses, there are many problems with this concept. Four seem to us to be of particular importance. The first is that there is no agreed definition of purging disorder. In many ways this is a minor problem that could easily be resolved. The second concerns the relationship between purging disorder and BN. Many people with purging disorder have subjective bulimic episodes (subjective binges) and are therefore very similar to people with true BN. Keel recognized this early on when she termed the state “subjective BN” rather than purging disorder.^21^ Given the debate over the significance of the distinction between objective and subjective binges,^21^ let alone the difficulty making it, Keel’s original term was apt as it could be argued that these cases might be better incorporated within BN. The third problem is related to the second. Purging may be “compensatory” or “noncom-pensatory”,^22^ a clinically important distinction that is not made in the literature on purging disorder. Compensatory purging is the use of purging to mitigate the effects on weight of specific episodes of perceived or actual overeating. If purging is compensatory it is linked to overeating episodes, follows them, and only occurs when they occur. Noncompensatory purging is when purging functions more as a “routine” form of weight control, akin to dietary restraint, in which case the link with episodes of overeating is not so close. Compensatory purging does not generally need to be addressed in treatment as measures to tackle binge eating remove compensatory purging as well.^23^ This is not the case with noncompensatory purging which tends to persist even if binge eating ceases. It therefore needs to be addressed in its own right.^23^ The fourth problem is the most important one. There is no evidence that the diagnosis of purging disorder has any prognostic or therapeutic implications. There are no data to suggest that patients with purging disorder differ from other EDNOS patients, or patients with BN, in either their course or response to treatment. For this reason we believe that it would be premature to recognize the concept. Doing so would certainly have the benefit of bringing to attention a subgroup of EDNOS cases but at the same time it might encourage neglect of the many others. After all, it is not just the purging disorder patients within EDNOS that have equivalent levels of psychopathology to patients with BN: this is true of EDNOS patients as a whole.^6^

We see the priority as being the collection of “transdiagnostic” data on treatment response and outcome. Such data are needed to derive clinically useful subdivisions within the overall category eating disorder. The study of complete transdiagnostic samples would be ideal as all forms of eating disorder would be represented. Again we are aware of only one study that has recruited such a sample and examined treatment response.^24^ More are needed.

## What to Do about the Classification of Eating Disorders in the Interim?

It is unlikely that there will be sufficient data available before DSM-V is finalized to answer the two questions that we have posed. Therefore an interim solution is needed. We favor the two-step one proposed by Fairburn and Bohn.^25^ Briefly, the first step involves accepting the current flawed classificatory scheme and expanding the diagnostic thresholds for AN and BN along the lines proposed in the literature. In other words it involves adding to the two established diagnostic concepts the “subthreshold” cases of AN and BN that exist within EDNOS. However, doing this only has a modest impact on the relative prevalence of EDNOS,^6^ and this is true even if BED is recognized as a diagnostic concept and its boundaries are expanded too.^6^ The explanation is that most cases of EDNOS are “mixed” in character with many of the clinical features of AN and BN being present but combined in subtly different ways from those seen in the two currently specified syndromes. What should be done about them? Fairburn and Bohn^24^ suggested that as the second step these cases be designated as belonging to a new all-embracing diagnostic category, perhaps termed “mixed eating disorder”. Admittedly this is something of a sleight of hand but it is one with a purpose; for this parsimonious and pragmatic solution would bring to attention the problems of all the remaining EDNOS patients while reducing the risk that new diagnostic subdivisions will be generated prematurely. It must be emphasized, however, that it is only an interim solution as it does not address the main limitations of the current classificatory scheme. Indeed, in some respects it simply highlights them.
